# A cluster randomized Hybrid Type III trial testing an implementation support strategy to facilitate the use of an evidence-based practice in VA homeless programs

**DOI:** 10.1186/s13012-015-0267-4

**Published:** 2015-05-28

**Authors:** David A. Smelson, Matthew Chinman, Sharon McCarthy, Gordon Hannah, Leon Sawh, Mark Glickman

**Affiliations:** 1 VA National Center on Homelessness Among Veterans, Bedford, MA USA; 2 VA Center for Healthcare Organization and Implementation Research, Bedford, MA USA; 3 Department of Psychiatry, University of Massachusetts Medical School, Worcester, MA USA; 4 VISN 4 Mental Illness Research and Clinical Center, VA Pittsburgh, Pittsburgh, PA USA; 5 RAND Corporation, Santa Monica, CA USA; 6 Department of Health Policy and Management, Boston University School of Public Health, Boston, MA USA

**Keywords:** Implementation support, Co-occurring disorders, Fidelity, Training, Technical assistance

## Abstract

**Background:**

The Housing and Urban Development-Veterans Affairs Supportive Housing (HUD-VASH) program is one of the largest initiatives to end Veteran homelessness. However, mental health and substance use disorders continue to reduce client stability and impede program success. HUD-VASH programs do not consistently employ evidence-based practices that address co-occurring mental health and substance use disorders. This paper presents a study protocol to evaluate the implementation of an evidence-based, co-occurring disorder treatment called Maintaining Independence and Sobriety Through Systems Integration, Outreach, and Networking—Veterans Edition (MISSION-Vet) in HUD-VASH using an implementation strategy called Getting To Outcomes (GTO).

**Methods/design:**

In three large VA Medical Centers, this Hybrid Type III trial will randomize case managers and their clients by HUD-VASH sub-teams to receive either MISSION-Vet Implementation as Usual (IU—standard training and access to the MISSION-Vet treatment manuals) or MISSION-Vet implementation augmented by GTO. In addition to testing GTO, effectiveness of the treatment (MISSION-Vet) will be assessed using existing Veteran-level data from the HUD-VASH data monitoring system. This project will compare GTO and IU case managers and their clients on the following variables: (1) fidelity to the MISSION-Vet intervention; (2) proportion of time the Veteran is housed; (3) mental health, substance use, and functional outcomes among Veterans; and (4) factors key to the successful deployment of a new treatment as specified by the Reach, Effectiveness, Adoption, Implementation, and Maintenance (RE-AIM) model.

**Discussion:**

This project is an important step for developing an implementation strategy to increase adoption of evidence-based practice use in VA homeless programs, and to further examine efficacy of MISSION-Vet in HUD-VASH. This project has important implications for program managers, policy makers, and researchers within the homelessness field. VA Central IRB approval for this study was granted in October 2011. The three sites were trained on MISSION-Vet and GTO in the first half of 2013. The first GTO planning meetings began after training occurred, between January 2013 and November 2013, across the three sites. The data collection—via a fidelity measure embedded into the VA Computerized Patient Record System—began as each site initiated MISSION-Vet, between April 2013 and January 2014.

**Trial registration:**

ClinicalTrials.gov: NCT01430741

## Background

Up to 80 % of Veterans who are homeless suffer from mental health and/or substance use disorders, which threatens housing stability and can result in higher rates of relapse, treatment dropout, poor community integration, and utilization of costly emergency and inpatient services [1]. Initially announced in 2009, a major goal for the Department of Veterans Affairs (VA) has been to end Veteran homelessness by the end of 2015 [2]. To sustain housing placements for Veterans who are formerly homeless, it is critical to integrate mental health and substance use treatments and wraparound supports [3]. While a number of treatment models have done this effectively, one evidence-based practice called Maintaining Independence and Sobriety through Systems Integration, Outreach, and Networking—Veterans Edition, or MISSION-Vet, was developed specifically for homeless and/or formerly homeless Veterans with co-occurring mental health and substance use disorders [4, 5]. However, incorporating MISSION-Vet into HUD-VASH—a national program that combines subsidized housing vouchers (individual Veterans receive “vouchers” for subsidized housing) from HUD and case management services from VA—can be a challenge as HUD-VASH has dramatically increased the number of Veterans served since 2008, resulting in larger caseload sizes and a need to prioritize housing placement [6]. This paper presents the protocol for a study testing strategies for proactively supporting the implementation of MISSION-Vet in HUD-VASH.

Despite a strong evidence base and support by the VA nationally for use of MISSION-Vet in HUD-VASH, wide-scale implementation has not occurred. MISSION-Vet has a brief, free, web-based training offered by the developers, as well as an accompanying Treatment Manual [4] and Consumer Workbook [5]. MISSION-Vet materials are available for free download on the National Center’s website (http://www.umassmed.edu/psychiatry/national-center-on-homelessness-among-veterans/products/). Further, MISSION-Vet shares some of the same techniques and treatment philosophies currently used by HUD-VASH, including Housing First (described below) [7]. Research using the Consolidated Framework for Implementation Research (CFIR) shows that national momentum, concrete tools, and compatibility with host sites are important factors that facilitate implementation. However, it cannot be assumed that prior efficacy of MISSION-Vet will automatically translate into success in HUD-VASH [8]. Systems do not always adopt new practices even when they are known to improve outcomes [9]. A number of studies have confirmed that factors at both the individual level (e.g., training, skills, efficacy, and involvement in decision making) and organizational level (organization size, climate, financial resources, and active support for evidence-based practices among the staff and administrators) predict successful implementation of evidence-based programs [10–15]. Existing implementation literature also suggests that active, innovative strategies are needed at both of these levels to encourage adoption, while passive approaches such as trainings alone does not result in change, as attendees often experience barriers to incorporating new practices into their work [15].

To facilitate the adoption of MISSION-Vet within HUD-VASH, we propose the use of the Getting To Outcomes (GTO) approach. GTO is both an implementation *model*—specifying the steps the staff should take when carrying out an evidence-based practice (EBP) such as MISSION-Vet—and an implementation *strategy*, providing ongoing implementation training, technical assistance, and data feedback to improve practitioners’ capacity to complete those steps [16]. While MISSION-Vet manuals promote fidelity to the treatment approach, GTO addresses system-level implementation barriers commonly found when providers begin to deliver a new treatment. It does so by building support among the staff in the use of the EBP, enhancing skills to complete needed tasks, and working collaboratively with the staff to tailor the EBP to the specific local site conditions. We received funding from the VA Quality Enhancement Research Initiative to compare MISSION-Vet implementation with and without GTO support in three large VA HUD-VASH sites.

### HUD-VASH program

HUD-VASH started as a 20-site demonstration project in 1992 and is now available at all VA medical centers. Recently, HUD-VASH has experienced two significant changes. First, the program was expanded to help support the goal of ending homelessness among Veterans. For example, between 2008 and 2014, HUD and the VA committed almost 70,000 additional vouchers, and the VA has funded over 3000 new case management positions [17]. Despite these new case manager hires, caseload sizes have increased for case managers. Second, HUD-VASH has increasingly adopted the Housing First philosophy, which states that individuals do not need to demonstrate a set period of sobriety in order to be considered eligible for housing and case management services, nor does non-adherence with case management necessarily lead to loss of housing [7]. In HUD-VASH, ongoing case management services are intended to serve as critical wraparound support to assist Veterans in recovery from substance abuse and/or mental illness while they continue to receive housing benefits. However, a study of 36 HUD-VASH sites found that the types and intensity of supports provided to Veterans tended to decrease over their tenure in the program, with less emphasis on rehabilitation-oriented activities [6]. While HUD-VASH serves many Veterans with co-occurring disorders, the program does not routinely use an evidence-based treatment model to address both mental health and substance abuse issues, both of which can threaten housing stability [6, 18]. Therefore, this study uses GTO implementation support to encourage the use of MISSION-Vet within HUD-VASH.

## Methods

### Overview

This project is a Hybrid Type III, cluster randomized controlled trial [19] that compares MISSION-Vet Implementation as Usual (IU—standard training and access to the MISSION-Vet treatment manuals) to MISSION-Vet implementation augmented by GTO. This study is being carried out in HUD-VASH teams at three large VA Medical Centers over a 2-year period. Within each site, existing sub-teams (case managers and Veterans they serve) are the clusters that will be randomly assigned, which is particularly appropriate when desiring to lessen the risk of experimental contamination [20] of the implementation strategy. Blinding in the traditional sense—e.g., data collectors blinded to subjects’ treatment assignment—will not be possible; however, all data will be coming from secondary sources. As a Hybrid Type III trial, it will emphasize the test of an implementation strategy (GTO), while using VA’s existing HUD-VASH data monitoring system to also assess the effectiveness of the treatment (MISSION-Vet) [21] called *HOMES* (Homeless Operations, Management and Evaluation System). This design is appropriate when the implementation strategy has strong preliminary evidence, the EBP in question has solid evidence but could yield weaker outcomes in new or less controlled environments than was present in efficacy trials, and the EBP has “implementation momentum” [21]. These conditions are present for implementing MISSION-Vet in HUD-VASH, as will be shown below. The trial assesses three sets of variables: a) MISSION-Vet implementation (adoption, dose, reach, fidelity), b) outcomes among HUD-VASH Veterans (mental health and substance use, community functioning, housing), and c) MISSION-Vet implementation barriers and facilitators organized by the CFIR.

### MISSION-Vet clinical intervention

MISSION-Vet is a flexible, integrated, co-occurring disorder treatment model rooted in the Health Belief Model [22] and is accompanied by a Treatment Manual [4], which serves as a how-to guide that describes MISSION-Vet’s core components, suggestions for service delivery, and includes a number of appendices with additional didactic materials. Optimally, each Veteran receives about 2.5 h of manualized services a week from a case manager and peer specialist team. The core service components of MISSION-Vet are critical time intervention (CTI), integrated co-occurring mental health and substance use disorder treatment using dual recovery therapy (DRT) delivered by a case manager, and peer support delivered by a peer specialist.

CTI [23], MISSION-Vet’s core intervention, is a time-limited assertive case management model intended to reduce the risk of homelessness by providing additional support to individuals with mental illness during the transition from institutions (e.g., inpatient psychiatry units, residential treatment programs, and homeless shelters) to community living. The assertive outreach in CTI is consistent with the role of case managers in HUD-VASH. Unlike HUD-VASH, however, CTI services are delivered in three phases of decreasing intensity: Transition to community, try-out, and transfer of care. In the transition to community phase, services are intended to reinforce community living. In the try-out phase, the case manager/peer specialist team begins to reduce service intensity to help the Veteran test and readjust the community-based support systems to fill any gaps. Visits in the transfer of care phase are used to fine-tune the connections established with community-based resources.

MISSION-Vet supplements CTI with 13 DRT [24–26] sessions delivered by the case manager. These sessions are highly structured, include the use of motivational interviewing [27] and relapse prevention [28] techniques, and specifically target the co-occurring mental health and substance abuse issues commonly facing homeless Veterans. In addition to the 13 DRT sessions, peer specialists deliver 11 structured psycho-educational sessions that are designed to empower Veterans to plan for a life of stability, sobriety, and community integration. Peer specialists give out the MISSION-Vet Consumer Workbook [5] at the onset of treatment to promote treatment engagement. Designed to serve as a compliment to the Treatment Manual, the Consumer Workbook helps Veterans integrate DRT and peer support concepts and increase their engagement in outpatient services through homework assignments, readings, and checklists.

In addition to CTI, DRT, and peer support, the MISSION-Vet Treatment Manual provides case managers/peer specialists with information regarding employment supports [29] and trauma-informed services [30], both of which have been found to be effective when used with the target population. While each component of MISSION-Vet has demonstrated efficacy on its own, when integrated using the MISSION-Vet platform, they work synergistically to increase treatment engagement, improve mental health and substance abuse outcomes, and reduce ER visits, re-hospitalizations, and recurring homelessness [31–34].

Besides the MISSION-Vet Treatment Manual and Consumer Workbook, MISSION-Vet also includes a structured webinar training found in previous studies to be successful in conveying needed information about the model (Smelson et al., unpublished manuscript). These 2-h trainings provide an overview of the MISSION-Vet treatment model and offer an introduction to the developed materials (manuals and fidelity measure). The training also covers the role of the supervisor whose job is to provide ongoing consultation ensuring fidelity to MISSION-Vet. This training is part of the IU strategy, along with key information on how to access and use the MISSION-Vet Treatment Manual and Consumer Workbook.

### GTO implementation model and strategy

GTO strengthens the knowledge, attitudes, and skills practitioners need to carry out evidence-based programs. GTO does this by posing a series of steps practitioners should follow in order to obtain positive results and then provides practitioners with the guidance necessary to complete those steps with quality (i.e., to perform each task as close to the ideal as possible). According to GTO, “carrying out” an evidence-based program involves much more than service delivery to patients, and the steps roughly correspond to three general areas: (1) planning—e.g., developing goals and performance targets, ensuring the staff are trained in the evidence-based program; (2) implementation—e.g., monitoring program activities, maintaining adherence to an evidence-based program model, supervision; and (3) self-evaluation—e.g., tracking patient outcomes, using data to improve program operations. All of these steps are designed to be logically linked so that the goals and performance targets are linked to program activities that will meet those targets, which in turn are linked to process and outcome measures to assess if the targets are being met, which in turn is linked to quality improvement activities that makes use of the process and outcome data. For all these areas, the guidance from the GTO approach comes in tools (GTO Implementation Handbook), face to face training for program (i.e., HUD-VASH) staff, and ongoing technical assistance provided by a GTO representative who meets regularly with the staff to help complete the needed steps. The goal is to work with leadership and staff to integrate the practices GTO targets into routine operations, closing the gap between research and practice.

GTO draws from multiple theories. It [16] is an operationalization of empowerment evaluation theory [35], which states that positive results are more likely when evaluators collaborate with program implementers and provide them with the tools and opportunities to evaluate and improve outcomes themselves. Consistent with social cognitive theories of behavioral change [36, 37], GTO training and technical assistance (TA) enhances knowledge about GTO-related activities, which improves attitudes towards these activities, improves execution of GTO-related behaviors, and supports strong implementation of EBPs [38] and outcomes.

The GTO implementation strategy is also grounded in implementation theory, for example, operationalizing the CFIR to ensure that all the major domains that influence implementation are considered [16]. Intervention characteristics (e.g., evidence strength and quality, relative advantage, complexity, adaptability), the first domain, can influence whether practitioners adopt an intervention. Without adaptation, many interventions are a poor fit. GTO helps practitioners tailor interventions to fit with their target population, organization, and broader community. The next three domains of the CFIR comprise the outer setting (e.g., broader social, political, and economic context including policies, incentives, and resources), inner setting in which the intervention is implemented (e.g., context of the specific organization or group implementing the intervention including the structural characteristics, relationships, and implementation readiness), and the characteristics of the individuals involved (e.g., knowledge, skills). In this study, the inner setting is the HUD-VASH team, the outer setting is the broader VA, and the individuals involved are the HUD-VASH staff. To create capacity and conditions for successful implementation of programs, the GTO implementation strategy targets both individual staff and its leadership (it is beyond GTO’s scope to specifically alter the broader social, political, and economic context of the outer setting). The active change process designed to facilitate individual and organization use of the intervention as designed is the last domain in CFIR. GTO proactively engages both the individual and program levels to establish a systematic implementation process.

A great deal of research has been done to examine GTO’s effectiveness. With practitioners of drug prevention programs, GTO has been found to improve the capacity of individual practitioners and the performance of prevention programs in both quasi-experimental and experimental trials. In addition to homelessness, GTO has been adapted to a number of other content areas including drug prevention, underage drinking prevention, teen pregnancy prevention, and positive youth development [16, 39–41]. Since its inception in 2004, more than 100,000 visits have been made to the website where the GTO model is described and over 100,000 GTO Implementation Handbooks have been downloaded (www.rand.org/gto).

### Participation sites and recruitment

#### Site composition

This study includes three large HUD-VASH sites: site A (450 HUD-VASH vouchers and 18 case managers), site B (850 HUD-VASH vouchers and 27 case managers), and site C (810 HUD-VASH vouchers and 24 case managers), for a total of 2110 HUD-VASH vouchers and 69 case managers. Case manager and voucher numbers were based on a point in time and are expected to change as new vouchers are issued over time. Each site has naturally occurring sub-teams of case managers (sites A, B, and C each has two clusters), which will be randomized with equal probability to IU or GTO by the team statistician using a random number generator. We chose to randomize case managers within each of these sites because it holds constant the variation due to site-level characteristics, funding streams, regulations, data collection activities, and political climates.

During the planning of the study, the three HUD-VASH programs were selected because they were similar across many key factors. For example, the number of vouchers allocated to each site is close to 500 with 85–95 % of these vouchers in use at each site. HUD-VASH case managers at each site are typically expected to have contact with each Veteran at least once per month—with a goal of contacting the Veteran at the Veteran’s residence once he/she has been housed. While some Veterans need more frequent contact, no Veteran gets less than this amount at any site. Among the Veterans in HUD-VASH at these sites, the VA Homeless Network Coordinators report approximately 90 % having substance use and/or mental health diagnoses. Additionally, each of these sites reports that approximately 75 % of Veterans in HUD-VASH receive other services at the VA or in the community beyond standard HUD-VASH case management services.

#### Recruitment

The subjects in this study are the HUD-VASH case managers although data about the Veterans on their caseload will also be collected. All case managers were invited to participate and consented before randomization by the study staff. The two sub-teams from each site that were assigned to GTO or IU have approximately equal numbers of case managers and vouchers, although the exact numbers shift frequently due to staff turnover and Veteran dropout, graduation, and the issuing of new vouchers. Regardless, consistent with cluster random assignment, each case manager is linked to a certain sub-team (and thus study condition), and each Veteran is in turn linked to a specific case manager. If sites decide to add new staff to a sub-team, that case manager would then automatically be assigned to the condition of that sub-team.

All HUD-VASH case managers in both groups will be invited to the webinar training on MISSION-Vet. After MISSION-Vet begins, case managers will be responsible for engaging their Veterans into MISSION-Vet services. Any Veteran participating in HUD-VASH at each of these three sites is eligible to receive MISSION-Vet services. Through training and TA, case managers and peer specialists will be guided to follow the recommended inclusion and exclusion criteria of MISSION-Vet: (1) has a current substance abuse or dependence disorder and a co-occurring mental illness and (2) is willing to participate in MISSION-Vet services. However, the decision with whom to deliver MISSION-Vet will ultimately be made by the case managers. MISSION-Vet takes about 1 year to fully deliver; therefore, Veterans will be invited to take part in MISSION-Vet in the first year of the 2-year intervention period. The study received a waiver of consent to use client-level data already being collected as part of the homeless data program management system of HUD-VASH.

### GTO implementation strategy to support MISSION-Vet implementation

The GTO implementation strategy is a capacity-building system that, at each site, consists of a “GTO Planning Team” of HUD-VASH staff (led by a designated point of contact) who will use the GTO process to plan MISSION-Vet and the GTO TA staff person (Dr. McCarthy, in Pittsburgh). The key components of this capacity-building system are training and written tools, TA, and the provision of tailored feedback based on MISSION-Vet service data. In particular, the type of TA provided in GTO is “facilitation,” a consultation method that emphasizes change in work practices through encouragement and action promotion [42, 43]:

1. *Training*—In month 5, the Study Team (PI Smelson, co-PI Chinman, TA staff McCarthy) will hold a training with the staff and leadership at each site. We will provide the standard MISSION-Vet training for the HUD-VASH staff followed by a 6-h training on how to use GTO to plan, implement, evaluate, and conduct quality improvement on MISSION-Vet. The MISSION-Vet training covers the MISSION-Vet Treatment Manual, Consumer Workbook, fidelity template, and other essential MISSION-Vet resources [4, 5], as well as core components and evaluation findings of MISSION-Vet. The GTO training involves walking the HUD-VASH staff through GTO’s model of planning, implementation, and self-evaluation, as applied to MISSION-Vet.
2. *GTO technical assistance and tools*—With guidance from the TA provider, each HUD-VASH site will use several GTO-based tools to plan MISSION-Vet that were specifically developed for homeless staff as part of the pilot project at the Pittsburgh VA Homelessness Center in the manual *Getting To Outcomes in services for homeless Veterans*: *10 steps for achieving accountability* [44]. GTO is meant to be a tiered system of support, with the TA staff person meeting by phone, bi-weekly with each site’s GTO Team, and then receiving their own support via weekly meetings with the PI and co-PI, who are experts in MISSION-Vet and GTO, respectively. This tiered model has been used in previous GTO projects with success [45]. Using the written tools, initial meetings of this group will focus on setting goals and performance targets, planning on how to tailor MISSION-Vet for HUD-VASH sites, identifying any gaps in skills required by MISSION-Vet, and arranging additional training. The TA staff person (Dr. McCarthy) will visit each site at least once a year.
3. *MISSION*-*Vet service tracking*—Prior to the start of MISSION-Vet implementation and during initial GTO planning with each team, the study staff will work with each site to integrate a MISSION-Vet “fidelity note template” into their local VA electronic medical record (called Computerized Patient Record System or CPRS). Each time the HUD-VASH staff delivers MISSION-Vet, they will use the template to record which aspects of MISSION-Vet were delivered, generating a clinical note. We will then extract this data and create feedback reports for each site, to be discussed in GTO team meetings once a month in order to stimulate quality improvement efforts.

### Measures, procedures, and analyses by study aim

All of the below measures will be collected for 3 years: during the 2-year GTO period of support and 1 year after that support ends to assess sustainability (see aim 4 below).

#### Aim 1: compare MISSION-Vet fidelity between GTO and IU groups

*Data source*. Veterans served by case managers in the GTO and IU groups.

*Measures and data collection.* The MISSION-Vet Fidelity Measure tracks all the core elements of the MISSION-Vet treatment model, including CTI, DRT, peer support, vocational supports, and trauma-informed care for each individual Veteran. The fidelity index consists of 78 items assessing the presence or absence of certain activities within MISSION-Vet. Taking the responses from all non-missing items, we will compute a fidelity score for each Veteran. The data will be extracted from the VA Computerized Patient Record System (CPRS) on all HUD-VASH Veterans served by case managers in the IU or GTO conditions, at each of the three HUD-VASH sites.

*Hypothesis.* We expect that Veterans served by case managers who were in the GTO group will receive MISSION-Vet with greater fidelity compared to those who are in the IU group.

*Data analysis.* We will fit cross-sectional linear random effects (to account for clustering) models for the fidelity score, measured at 12 months post-baseline.

#### Aim 2: compare the effect on the proportion of time the Veteran is housed between GTO and IU groups

*Data sources*. Veterans served by case managers in the GTO and IU groups.

*Measures and data collection*. For each Veteran entering HUD-VASH, a case manager must complete a HUD-VASH Referral Worksheet that contains the program’s first assessment of days of homelessness and housing. Then, case managers provide data every 3 months on the number of days housed in the last 90 days using the Housing Progress Report form. Case managers are also required to report when a Veteran loses their housing voucher each month. These reports are stored in national database called HOMES (Homeless Operations, Management and Evaluation System).

*Hypothesis*. We expect that Veterans assigned to case managers in the GTO group, who receive MISSION-Vet, will have more days housed in the prior 3 months compared to Veterans who receive MISSION-Vet from case managers in the receiving IU group.

*Data analysis*. Again, we will fit repeated measures linear random effects models in this analysis. As a secondary analysis, we will perform a *z* test for the comparison of the proportion of Veterans at the end the study who have retained their housing (versus lost their voucher) between GTO versus IU.

#### Aim 3: compare mental health, substance use, and functional outcomes among Veterans served by HUD-VASH case managers in GTO and IU groups

*Data sources*. Veterans served by case managers in the GTO and IU groups.

*Measures and data collection*. In addition to housing, the HUD-VASH staff complete data collection forms for HOMES on four outcomes each month. HOMES requires case managers to make separate ratings on mental health, substance abuse, and associated problems for each Veteran using components of the Addiction Severity Index [46] as well as the Global Assessment of Functioning (GAF) [47].

*Hypothesis*. We expect that Veterans served by HUD-VASH case managers supported by GTO, who received MISSION-Vet, will have improved mental health, substance use, and functional outcomes compared to Veterans served by HUD-VASH case managers in the IU group.

*Data analysis*. We will again fit our repeated measures linear random effects models to the response variables mentioned above.

#### Aim 4: analyze MISSION-Vet implementation using the factors specified by the implementation model RE-AIM (Reach, Effectiveness, Adoption, Implementation, and Maintenance)

RE-AIM [48] states that successful deployment of an intervention is mediated by the following factors: *Reach*—the proportion of individuals reached by the intervention; *Effectiveness*—the efficacy of the program under ideal circumstances; *Adoption*—the proportion of patients that used the intervention; *Implementation*—the extent the intervention was well-implemented; and *Maintenance*—whether the intervention was maintained. While GTO provides guidance for successful implementation, the RE-AIM model provides a heuristic to judge the success of that implementation.

*Data sources*. All aspects of the RE-AIM framework will be informed by data from case managers in the IU and GTO groups and the Veterans they serve.

*Measures and data collection*. Each RE-AIM measure below, using data collected from aims 1–3, will be calculated for both the IU and GTO groups:

- *Reach*—Obtained from aim 1, the Reach measure will be the percent of all eligible Veterans who receive MISSION-Vet with adequate fidelity (defined as the endorsement of at least 75 % of the items on the MISSION-Vet Fidelity measure).
- *Effectiveness*—Obtained from aim 3, effectiveness will be the percentage of all eligible Veterans *who receive MISSION*-*Vet with adequate fidelity* who improve on each outcome in an amount consistent with a statistically significant effect.
- *Adoption*—The proportion of case managers who have more than 50 % of their eligible Veterans engaged in MISSION-Vet will be judged to have “adopted” MISSION-Vet as a standard protocol for Veterans with mental illness and co-occurring substance abuse disorders.
- *Implementation*—The extent to which the MISSION-Vet intervention was well implemented will come from aim 1 and be expressed as the percentage of eligible Veterans who actually participated in MISSION-Vet who had adequate fidelity (endorsement of at least 75 % of the items on the MISSION-Vet Fidelity measure).
- *Maintenance*—The degree to which the successful deployment of MISSION-Vet was maintained will come from data collected through the previous components of the RE-AIM model but extended a year beyond the end of the GTO support. Thus, this study will be able to assess whether MISSION-Vet maintains a high Reach, Effectiveness, Adoption, and Implementation between the end of the GTO support and the last data collection wave, which will be a year after the GTO support ends.

*Hypothesis*. We expect better results across all elements of the RE-AIM model in the GTO group than in the IU group.

*Data analysis*. The scores for R, E, A, and I will be calculated among the Veterans in both the GTO and IU groups during the 2-year period of GTO support. For *Reach*, we will perform a *z* test for the comparison of the proportion of Veterans at the end of the GTO period with adequate fidelity between GTO versus IU. The *Effectiveness* analysis will involve *t* tests comparing mean scores at the end of the GTO period for the proportion of time housed and homeless (on the logit scale), components of the ASI, and the GAF. For *Adoption*, we will compare the proportion of case managers through a *z* test who have “adopted” MISSION-Vet according to the specified measure. We will also perform a logistic regression of whether a case manager adopted MISSION-Vet as a function of study arm, one of the moderating variables, and their interaction, testing specifically for the interaction term. The analysis of *Implementation* will compare through a *z* test the proportion of Veterans who participated in MISSION-Vet who had a fidelity score of at least 75 % between the GTO versus IU groups.

*Maintenance*—The analyses described above for R, E, A, and I will be similarly applied to the data collected and summarized a year after the end of the GTO support period.

### Power analysis

We demonstrate that for aims 1, 2, and 3, we will have at least 80 % power to detect intervention effects at the 0.05 significance level (aim 4 is exploratory and thus we are not able to calculate power for that aim). Our power calculations were performed using the power analysis software G*Power 3.1.2 [49]. Previous studies examining the effect of GTO used different measures than in the current study. We therefore assumed effect sizes based on a previous study of the effect of GTO on capacity to carry out evidence-based drug abuse prevention programming. The measures used in that study were of provider knowledge (effect size = 0.21 after 2 years of GTO), attitudes (effect size = 0.38 at 1 year of GTO and 0.59 at 2 years of GTO), and skills (effect size = 0.07 at 1 year of GTO and 0.28 at 2 years of GTO) all related to the performance of practices associated with evidence-based prevention programming [16]. These measures are relevant because GTO will attempt to impact knowledge, attitudes, and skills in the proposed study as well, but in this case around the use of MISSION-Vet. We use these values as a rough guide for the effects we might expect to detect for the current study, along with conventional effect size interpretations.

For aim 1, we expect a total of 1604 Veterans to be available for the fidelity measure computations. This sample size assumes a reduction of 20 % from the initial (approximate) 2110 vouchers available due to Veterans not having co-occurring SUD and then another reduction of 5 % due to case manager refusal to participate. The latter reduction brings the number of case managers down from an initial estimate of 69 down to 66. Because the study is cluster-randomized by case manager, the sample size to detect effects is reduce by a “design effect” (Deff). The formula for the design effect is given by Deff = 1 + (*m* − 1) ICC where *m* is the average number of Veterans in the study per case manager and the ICC is the intra-class correlation. The ICC of MISSION-Vet + GTO effects clustered by case managers is unknown but likely to be fairly limited as GTO is a system-wide intervention. To be conservative, we use a conventionally large value of ICC = 0.15 [50]. Based on the information above, the value of *m* is approximately *m* = 1604/66 = 24.3. Thus, the design effect assumed is Deff = 1 + (*m* − 1) ICC = 1 + (24.3 − 1) (0.15) = 4.495. Therefore, the effective sample size for aim 1 controlling for clustering is 1604/4.495 = 356 = 265 Veterans. For an unclustered design with an available sample of 356 Veterans randomized to two study arms, the smallest detectable effect size (ES) at the 0.05 significance level with 80 % power assuming a two-sample *t* test is 0.298. While this effect is of comparable or larger size to those in the drug abuse prevention study described above, this effect size, according to [51], is considered conventionally to be between small (ES = 0.2) and medium (ES = 0.5). Therefore, our sample size seems to be sufficient to detect a small-to-medium effect size. The actual data analyses will control for socio-demographic and other important confounders, so in fact we expect greater power to detect fidelity differences due to MISSION-Vet + GTO.

For aims 2 and 3, we will focus on the subsample of Veterans in the study for whom we have 12 months of data collected over consecutive 3-month intervals. Because these Veterans serve as their own controls relative to time, they provide the greatest power in detecting the effect of MISSION-Vet + GTO. With the same design effect as in aim 1, the effective sample size available for aims 2 and 3 controlling for clustering is 356 Veterans. For a linear repeated measures model with two treatment groups and five measurements per unit (that is, one every 3 months for a year, including baseline), and assuming a sample of 356 units to randomly divide approximately equally between the two groups, the time-by-intervention effect size that can be measured with 80 % power is 0.082. By convention, this is considered a very small effect size, and it is also small compared to the ones found above in the drug abuse prevention study. We therefore anticipate detecting effects with sufficient power for aims 2 and 3.

### Design challenges

An important design issue is whether to randomize at the site, provider, or Veteran level. We decided to randomize at the HUD-VASH case manager level for several reasons. First, the analysis of national HUD-VASH data showed that the HUD-VASH site was the strongest predictor of housing stability [6]. Therefore, it was important to hold the site constant by having IU and GTO conditions equally represented within each of the sites. Further, we account for the effects of case manager clustering in our statistical analyses. Second, we believe that the possible contamination that may result between IU and GTO conditions being present at the same site is manageable. In the past studies of GTO, contamination did occur but was moderate (across multiple programs, mean = 0.5 to 1.0 on a 6-point index of possible GTO activities in which one could engage) [16]. We have however, purposely selected large sites in an effort to minimize the contamination as it is believed that we can minimize discussion of GTO implementation strategies more easily among non-GTO case managers in larger sites. To minimize contamination further given its potential confound, we decided to use naturally occurring HUD-VASH sub-teams at each site so that team meetings and other routine discussions between case managers are handled in separate IU and GTO “teamlets.” Other limitations of the study include staff turnover and changes in policy directives. Regarding staff turnover, we anticipate some level of turnover, and our power analyses show that we have a sufficient sample. Furthermore, new staff in the IU sites will receive training on MISSION-Vet. As for changes in the program directives, we acknowledge that this is also a limitation as there is always ongoing tension between research and the need for patient care services to make and respond to new directives to end homelessness. However, our design—comparing an organizational intervention like GTO to usual implementation strategies (regardless of how they may change)—is therefore a genuine test of the impact of the GTO intervention (and MISSION-Vet as well) in real-world conditions. Thus, the findings that are produced may have greater ecological validity—or relevance to real world conditions, where circumstances do change. In addition, even if the VA did make changes to HUD-VASH, the changes would apply evenly to all HUD-VASH sites and all HUD-VASH case managers and thus be equal across IU and GTO conditions. Further, we would hypothesize that GTO would be able to help HUD-VASH case managers incorporate the changes for improved Veteran impact better than case managers assigned to the IU condition.

### Trial status

Central IRB approval for this study was granted in October of 2011. The three sites were trained on MISSION-Vet and GTO in the first half of 2013. The first GTO planning meetings began directly after training occurred, between January 2013 and November 2013 across the three sites. The data collection—via a fidelity measure embedded into the VA Electronic Medical Record System—began as each site initiated MISSION-Vet, between April 2013 and January 2014.

## Discussion

As noted above, research evidence and passive dissemination strategies often do not change clinical practice or increase the adoption of new practices. MISSION-Vet has demonstrated evidence, yet has not been widely adopted despite encouragement from the VA nationally. This project intends to test whether a comprehensive implementation strategy—Getting To Outcomes—can aid in MISSION-Vet’s implementation in HUD-VASH and secondarily to evaluate the effectiveness of the MISSION-Vet treatment. Further, this project will yield important lessons about *how* and *under what conditions* GTO support aids MISSION-Vet implementation. The test will be challenging as HUD-VASH teams are under increasing pressure to focus on housing placement to meet Obama’s administration pledge to end homelessness. However, the case managers randomized to receive GTO will have a number of additional supports to assist with the implementation of the MISSION-Vet model within HUD-VASH. If successful, GTO could be used as a model to more widely support MISSION-Vet implementation or the implementation of other evidence-based practices. In addition, study results will continue to evaluate the efficacy of MISSION-Vet for the treatment of homeless Veterans with co-occurring disorders.

This project is registered at ClinicalTrials.gov with number NCT01430741 (URL: https://clinicaltrials.gov/ct2/show/study/NCT01430741?term=sdp+11-240&rank=1).

## Abbreviations

GTO: Getting To Outcomes
HUD-VASH: Housing and Urban Development-Veteran Affairs Supportive Housing
MISSION-Vet: Maintaining Independence and Sobriety through Systems Integration, Outreach, and Networking—Veterans Edition
